# Convalescent Plasma for Preventing Critical Illness in COVID-19: a Phase 2 Trial and Immune Profile

**DOI:** 10.1128/spectrum.02560-21

**Published:** 2022-02-23

**Authors:** Jeffrey M. Sturek, Tania A. Thomas, James D. Gorham, Chelsea A. Sheppard, Allison H. Raymond, Kristen Petros De Guex, William B. Harrington, Andrew J. Barros, Gregory R. Madden, Yosra M. Alkabab, David Y. Lu, Qin Liu, Melinda D. Poulter, Amy J. Mathers, Archana Thakur, Dana L. Schalk, Ewa M. Kubicka, Lawrence G. Lum, Scott K. Heysell

**Affiliations:** a Division of Pulmonary & Critical Care Medicine, Department of Medicine, University of Virginia grid.412587.dgrid.27755.32 School of Medicine, Charlottesville, Virginia, USA; b Division of Infectious Diseases & International Health, Department of Medicine, University of Virginia grid.412587.dgrid.27755.32 School of Medicine, Charlottesville, Virginia, USA; c Division of Laboratory Medicine, Department of Pathology, University of Virginia grid.412587.dgrid.27755.32 School of Medicine, Charlottesville, Virginia, USA; d Division of Cardiology, Department of Medicine, University of Virginia grid.412587.dgrid.27755.32 School of Medicine, Charlottesville, Virginia, USA; e College of Arts and Sciences, Cornell University, Ithaca, New York, USA; f The Wistar Institute, Philadelphia, Pennsylvania, USA; g Division of Hematology and Oncology, Department of Medicine, University of Virginia grid.412587.dgrid.27755.32 School of Medicine, Charlottesville, Virginia, USA; Karolinska Institutet

**Keywords:** SARS-CoV-2, antibodies, respiratory failure, COVID-19, convalescent plasma

## Abstract

The COVID-19 pandemic caused by the severe acute respiratory syndrome coronavirus 2 (SARS-CoV-2) is an unprecedented event requiring frequent adaptation to changing clinical circumstances. Convalescent immune plasma (CIP) is a promising treatment that can be mobilized rapidly in a pandemic setting. We tested whether administration of SARS-CoV-2 CIP at hospital admission could reduce the rate of ICU transfer or 28-day mortality or alter levels of specific antibody responses before and after CIP infusion. In a single-arm phase II study, patients >18 years-old with respiratory symptoms with confirmed COVID-19 infection who were admitted to a non-ICU bed were administered two units of CIP within 72 h of admission. Levels of SARS-CoV-2 detected by PCR in the respiratory tract and circulating anti-SARS-CoV-2 antibody titers were sequentially measured before and after CIP transfusion. Twenty-nine patients were transfused high titer CIP and 48 contemporaneous comparable controls were identified. All classes of antibodies to the three SARS-CoV-2 target proteins were significantly increased at days 7 and 14 post-transfusion compared with baseline (*P* < 0.01). Anti-nucleocapsid IgA levels were reduced at day 28, suggesting that the initial rise may have been due to the contribution of CIP. The groups were well-balanced, without statistically significant differences in demographics or co-morbidities or use of remdesivir or dexamethasone. In participants transfused with CIP, the rate of ICU transfer was 13.8% compared to 27.1% for controls with a hazard ratio 0.506 (95% CI 0.165–1.554), and 28-day mortality was 6.9% compared to 10.4% for controls, hazard ratio 0.640 (95% CI 0.124–3.298).

**IMPORTANCE** Transfusion of high-titer CIP to non-critically ill patients early after admission with COVID-19 respiratory disease was associated with significantly increased anti-SARS-CoV-2 specific antibodies (compared to baseline) and a non-significant reduction in ICU transfer and death (compared to controls). This prospective phase II trial provides a suggestion that the antiviral effects of CIP from early in the COVID-19 pandemic may delay progression to critical illness and death in specific patient populations. This study informs the optimal timing and potential population of use for CIP in COVID-19, particularly in settings without access to other interventions, or in planning for future coronavirus pandemics.

## INTRODUCTION

Passive antibody infusion was the first immunotherapy dating back to the 1890s for the treatment of infectious diseases before the development of antibiotics (1–3). Experience from prior outbreaks with other coronaviruses, such as SARS-CoV-1, shows that such convalescent immune plasma (CIP) contains neutralizing antibodies to the virus (4). The use of CIP or hyperimmune immunoglobulin-containing specific antiviral antibodies has been safe and effective with other respiratory viral infections (5); and in the most recent Ebola outbreak, treatment with neutralizing antibodies improved survival when compared to pharmacologic inhibition (6).

In March 2020, the US Food and Drug Administration (FDA) authorized CIP for compassionate use, in April it approved an expanded access program (EAP) for use in hospitalized patients, and in August the FDA granted CIP emergency use authorization (EUA). CIP remains one of the few therapies to have EUA for hospitalized patients along with the antiviral remdesivir and the selective inhibitor of Janus kinase 1 and 2, baricitinib (7). Yet over the course of the pandemic, treatment trials in COVID-19 have yielded a range of discrepant results, notably with the recent Solidarity trial showing no benefit of remdesivir (8), highlighting the need for further studies.

CIP is commonly considered as a bridge to specific monoclonal antibody therapies. While such therapies have demonstrated efficacy in outpatients with COVID-19 (9, 10), a study of the monoclonal antibody bamlanivimab did not show sustained recovery in hospitalized patients, and the study was stopped early for futility (11). The failure of a monoclonal antibody in this population suggests that CIP containing polyclonal antibodies targeting multiple antigens may have a clinical advantage. Given the rapidity with which CIP can be mobilized, studies that confirm safety and efficacy can inform its role in future coronavirus pandemics (12).

Although the rapid expansion of compassionate use of CIP for hospitalized patients in the US early in the coronavirus disease 2019 (COVID-19) pandemic made it challenging to conduct controlled trials (13), this formal phase II was successful in enrolling patients on an FDA, IRB approved clinical study (NCT04374565). The primary objectives of this phase II single-arm study were to determine if early administration of high titer SARS-CoV-2 CIP to adults hospitalized with respiratory symptoms from COVID-19 would be safe, and whether it would affect pre-specified primary endpoints of transfer to the intensive care unit (ICU) and 28-day mortality. Pre-specified secondary endpoints included clinical as well as immunologic endpoints, such as to determine the kinetics of the development of specific antibody titers to SARS-CoV-2 viral proteins and determine correlation with specific antibody levels and the persistence of virus.

## RESULTS

### Clinical outcomes.

Thirty-two participants were enrolled to reach the target of 29 evaluable patients who were transfused with CIP. One participant was discharged within a day of enrollment and prior to receipt of CIP, while two other participants signed consent but later declined CIP. Among 149 potential controls who were screened, 48 met eligibility (Table 1). Participants who received CIP and controls were well-balanced, without statistically significant differences in demographics or co-morbidities. The median age (Q1, Q3) of participants and controls were 61.1 (51.7, 66.9) years and 65.3 (49.2, 78.4) years, respectively (*P* = 0.13). Fourteen (48%) participants were female compared to 30 (63%) controls (*P* = 0.24). People of Black race or Hispanic ethnicity comprised the majority in both groups, including 19 (66%) participants and 36 (75%) controls, reflecting regional COVID-19 demographics. The most common comorbidity was hypertension, present in 17 (59%) participants and 31 (65%) controls (*P* = 0.63). The two groups were similar both in initial severity of illness (with no difference in WHO score at admission) and in time from symptom onset to admission (5 days on average in each group) (Table 1). The controls were drawn from the same time-period as the trial enrollment plus 4 weeks preceding trial opening as described in the Methods, and importantly case counts and in-hospital mortality based on data extraction from the clinical data warehouse did not vary significantly over this time period (Fig. S1 in the supplemental material, *P* = 0.45). Lastly, both groups received similar treatment with dexamethasone, 15 (51.7%) participants and 21 (43.8%) controls (*P* = 0.64), and remdesivir, 9 (31.0%) participants and 15 (31.2%) controls (*P* > 0.99).

**TABLE 1:** Demographic and clinical characteristics among participants receiving convalescent plasma and controls

Characteristic	Convalescent plasma (*N* = 29)	Controls (*N* = 48)	*P*-value
Age yr, median (Q1, Q3)	61.1 (51.7, 66.9)	65.3 (49.2, 78.4)	0.131
Sex, female (%)	14 (48.3)	30 (62.5)	0.24
Race/ethnicity			
White Non-Hispanic (%) Hispanic (%) Black (%) Asian American Indian/Alaskan Native Other	8 (27.6) 12 (41.4) 7 (24.1) 0 0 2 (6.9)	10 (20.8) 14 (29.2) 22 (45.8) 0 0 2 (4.1)	0.26
Diabetes	12 (41.4)	19 (39.6)	>0.99
Hypertension	17 (58.6)	31 (64.6)	0.63
Asthma	2 (6.9)	5 (10.4)	0.71
COPD/emphysema	3 (10.3)	4 (8.3)	>0.99
World Health Organization Ordinal scale at admission^*a*^ Category 3 (%) Category 4 (%)	10 (34.5) 19 (65.5)	18 (37.5) 30 (62.5)	0.81
Admission laboratory values^*b*^			
Creatinine mg/dL, median (Q1, Q3)	1.1 (0.7, 1.4)	1.0 (0.7, 1.3)	0.27
White blood cell count k/μL, median (Q1, Q3)	6.5 (4.9, 7.6)	5.7 (4.5, 6.9)	0.333
Total lymphocyte count k/μL, median (Q1, Q3)	0.8 (0.7, 1)	0.9 (0.6, 1.4)	0.54
D-dimer ng/mL, median (Q1, Q3)	322 (241, 604)	301 (219, 454)	0.46
Ferritin ng/mL, median (Q1, Q3)	522 (319, 1075)	445 (257, 876)	0.27
C-reactive protein mg/dL, median (Q1, Q3)	9 (4.8, 13.5)	11.7 (3.6, 14.1)	0.74
BMI, median (Q1, Q3)	32.9 (31.6, 41.8)	33.6 (28.0, 37.6)	0.551
Days from first symptom onset to admission, median (Q1, Q3)	5 (3, 7)	5 (2, 8.5)	0.54

*^a^*Study enrolled only those with score 3 (hospitalized but no supplemental oxygen) and score 4 (hospitalized with oxygen by nasal prongs or mask but without high-flow or noninvasive ventilation).

*^b^*Normal laboratory values: Creatinine (available in all participants with convalescent plasma and controls) adult male 0.7–1.3 mg/dL and adult women 0.4–1.1 mg/dL; white blood cell count (available in all participants that received convalescent plasma and controls) 4.0-11.0 k/μL; absolute lymphocyte count 1.0–5.0 k/μL; d-dimer (available in 26 participants that received convalescent plasma and 39 controls) <243 ng/mL; Ferritin (available in 23 participants that received convalescent plasma and 37 controls) adult male 20–275 ng/mL and adult female 5–200 ng/mL; C-reactive protein (available in 23 participants that received convalescent plasma and 37 controls) <0.5 mg/dL. BMI control *n* = 42, BMI convalescent plasma *n* = 26.

Of the 29 participants that received CIP, 13 (45%) received their first transfusion within 24 h of admission, 14 (48%) within 48 h, and 2 (6%) within 72 h. One participant (3%) received only one unit of CIP, while the remainder received two units; in 5 (17%) participants, limited supply of available CIP necessitated administration of two units from different donors. All transfused units had detectable IgG to the spike protein, with a considerable range in titer (Table 2, Fig. S2 in the supplemental material).

**TABLE 2:** Immunological profile of the transfused convalescent plasma units^*a*^

Specific antibody	IgG	IgM	IgA
Anti-spike, median (min-max) (μg/mL for IgG, EU/mL for IgM and IgA)	7.7 (0.1–112.1)	3.0 (0–106.6)	2.9 (0–24.7)
Anti-RBD, median (min-max) (μg/mL for IgG, EU/mL for IgM and IgA)	2.7 (0.1–83.9)	2.9 (0–27.7)	2.6 (0–23.5)
Anti-nucleocapsid, median (min-max) (EU/mL for IgG, IgM, and IgA)	0.52 (0.0–8.67)	1.3 (0–10.0)	0 (0–2.3)

*^a^*All units were also screened by the commercial Abbott assay which measures IgG to nucleocapsid but is reported as signal to cutoff values. Per manufacturer recommendation, signal to cutoff values of 1.4 or greater were used to screen. Thirty-nine of 42 paired units screened had signal to cutoff values of 1.4 or greater, with range of transfused units from 2.29 to 9.33.

There were 24 adverse events among 11 participants (Table S1 in the supplemental material). Of the serious adverse events, 7 (29%) were grade 3 or above, but none were categorized as related to CIP transfusions. There were no events of transfusion-related acute lung injury (TRALI) or transfusion-associated circulatory overload (TACO).

**FIG 1:**
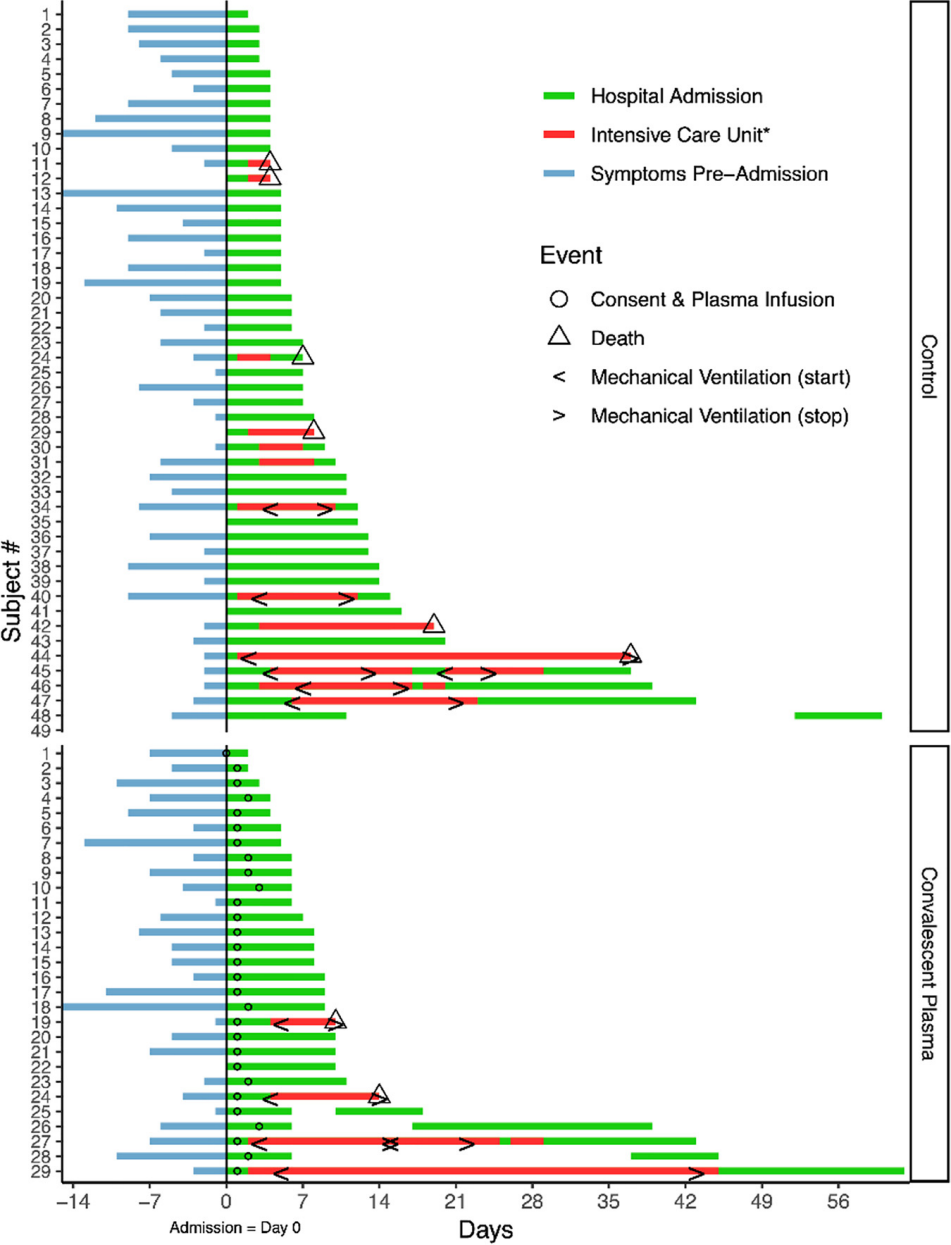
Swimmer plot depicting clinical timelines of CIP transfused participants and controls. Baseline was the day of index hospital admission. The blue line represents symptomatic days before admission; green lines represent admission to acute hospital care, with intensive care unit stays represented in red. Blank gaps between hospitalizations indicate the patient was discharged then readmitted within the 60 day follow up period. Circles show the date of plasma infusion; triangles indicate that the patient died; “< >” bracket time periods where the patient received mechanical ventilation. Participant 29 in the Convalescent Plasma group was discharged on day 60. *Pictured intensive care unit stays were indicated for higher levels of oxygen therapy including high-flow nasal cannula oxygen, mechanical ventilation, and/or extracorporeal membrane oxygenation.

**FIG 2:**
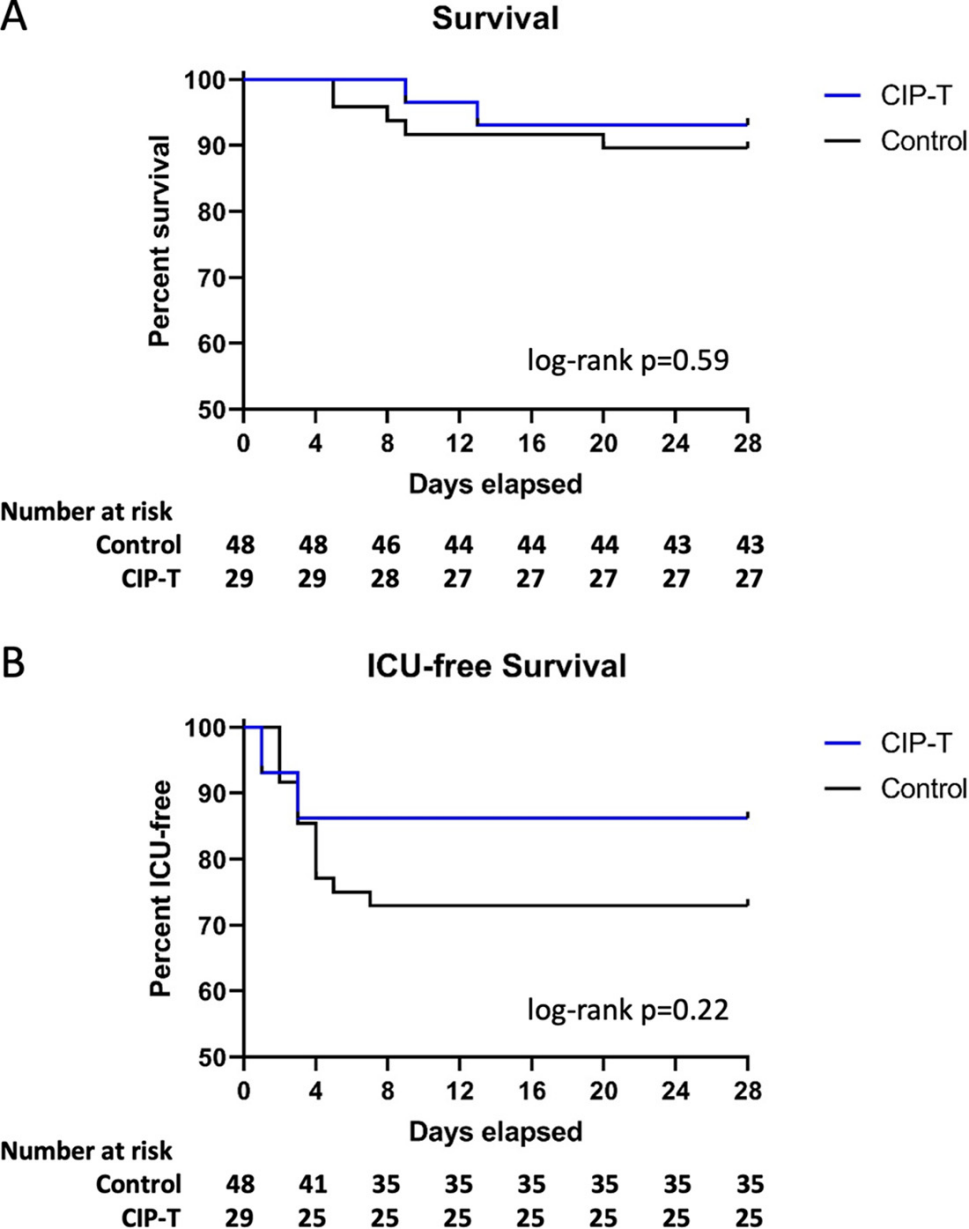
Effect of CIP on progression to critical illness and survival. Kaplan-Meier curves are shown comparing survival (A) and ICU-free survival (B) in CIP transfused patients vs control. Number of patients remaining at risk are listed along the bottom of each panel. Log-rank *P* values are listed.

The clinical course of each participant is visually represented in the swimmer’s plots depicted in Fig. 1. Kaplan-Meier curves for survival and ICU-free survival are represented in Fig. 2. We observed a non-statistically significant reduction in the primary endpoint of ICU transfer, with 14% of transfused patients ultimately requiring ICU transfer compared to 27.1% for controls (Fisher’s exact *P*-value = 0.258). The second primary endpoint of 28-day mortality was similarly non-significantly reduced in the study group at 6.9% compared to 10.4% in controls. A univariate Cox regression analysis for time-to-death (Table 3) showed a hazard ratio (HR) of 0.640 (95% CI 0.124–3.298). Due to the low event rate, no multivariate analysis was performed. Of the other variables tested, only age was significantly associated with mortality, with a HR of 1.103 (95% CI 1.047–1.162). With regard to ICU transfer rate, the univariate time-to-event analysis revealed a HR of 0.506 (95% CI 0.165–1.554) (Table 4). Univariate analyses again showed a weak but statistically significant association of ICU transfer with age, HR 1.03 (95% CI 1.000–1.061). On multivariate analysis (Table 5), the HR for CIP remained similarly non-significantly reduced at 0.501 (95% CI 0.145–1.739). In this analysis, dexamethasone use was found to be significantly associated with risk of ICU transfer (HR 3.376, 95% CI 1.045–10.909).

**TABLE 3:** Univariate Cox regression analysis for time to death by 28 days after entering study (*N* = 77)

Variables	Hazard ratio (HR)	*SE*	z	*P* > *z*	Lower 95% CI of HR	Upper 95% CI of HR
CIP	0.640	0.535	−0.530	0.593	0.124	3.298
Age	1.103	0.029	3.700	<0.001	1.047	1.162
Sex (F vs. M)	1.009	0.771	0.010	0.991	0.226	4.508
Hypertension	3.693	3.989	1.210	0.226	0.445	30.678
Diabetes	1.107	0.845	0.130	0.894	0.248	4.946
Remdesivir	0.868	0.726	−0.170	0.866	0.168	4.474
Dexamethasone	0.828	0.633	−0.250	0.805	0.185	3.702
BMI (*N* = 68)	0.919	0.059	−1.310	0.191	0.811	1.043
Obese (*N* = 68)	0.571	0.521	−0.610	0.540	0.095	3.419

**TABLE 4:** Univariate Cox regression analysis for time to ICU (*N* = 77)

Variables	Hazard ratio (HR)	*SE*	z	*P*-value	Lower 95% CI of HR	Upper 95% CI of HR
CIP	0.506	0.290	−1.190	0.234	0.165	1.554
Age	1.030	0.015	1.990	0.047	1.000	1.061
Sex (F vs. M)	1.058	0.522	0.120	0.908	0.403	2.781
Hypertension	1.091	0.554	0.170	0.864	0.403	2.949
Diabetes	0.946	0.466	−0.110	0.911	0.360	2.487
Remdesivir	1.242	0.631	0.430	0.669	0.459	3.360
Dexamethasone	2.344	1.190	1.680	0.093	0.867	6.342
BMI (*N* = 68)	1.023	0.026	0.880	0.381	0.973	1.075
Obese (*N* = 68)	1.619	1.045	0.750	0.456	0.457	5.736
WHO score (oxygen vs no oxygen)	1.044	0.530	0.080	0.932	0.386	2.823

**TABLE 5:** Multivariate Cox regression analysis for time to ICU (N = 77)

Variables	Hazard ratio (HR)	*SE*	z	*P*-value	Lower 95% CI of HR	Upper 95% CI of HR
CIP	0.501	0.318	−1.090	0.277	0.145	1.739
Age	1.035	0.018	2.000	0.046	1.001	1.070
Sex (F vs. M)	0.863	0.480	−0.270	0.790	0.290	2.566
Hypertension	0.709	0.410	−0.600	0.552	0.228	2.201
Diabetes	1.122	0.668	0.190	0.846	0.350	3.602
Remdesivir	0.642	0.387	−0.740	0.462	0.197	2.092
Dexamethasone	3.376	2.020	2.030	0.042	1.045	10.909
BMI (*N* = 68)	0.672	0.418	−0.640	0.523	0.199	2.276

Secondary clinical outcomes are summarized in Table 6. There was no significant effect of CIP on any of the clinical secondary endpoints.

**TABLE 6:** Secondary clinical endpoints

Secondary clinical endpoints	Convalescent plasma (*N* = 29)	Controls (*N* = 48)	*P*-value
Hospital length of stay, median (Q1, Q3)	8 (6, 10)	7 (4.75, 12.25)	0.987
ICU length of stay, median (Q1, Q3)	0 (0, 0)	0 (0, 2)	0.244
ICU-free days, median (Q1, Q3)	28 (28, 28)	28 (23.75, 28)	0.238
Ventilator-free days, median (Q1, Q3)	28 (28, 28)	28 (28, 28)	0.412
Need for renal replacement therapy, n (%)	1 (3.4%)	1 (2.1%)	>0.99
Need for extracorporeal membrane oxygenation (ECMO)	1 (3.4%)	0 (0.0%)	0.377

### Quantification of specific anti-SARS-CoV-2 antibodies.

**(i) Levels of specific antibodies in CIP.** The levels of specific IgG, IgM, and IgA to spike (S), receptor binding domain (RBD), and nucleocapsid (NC) were measured to determine the levels of each class of specific antibody in the CIP transfused (Table 2, Fig. S2, panel A, in the supplemental material). The quantities of IgG anti-NC in the transfused units as measured by the Abbott assay (S/CO) vs the specific ELISA are available in Fig. S2, panel B.

**FIG 3:**
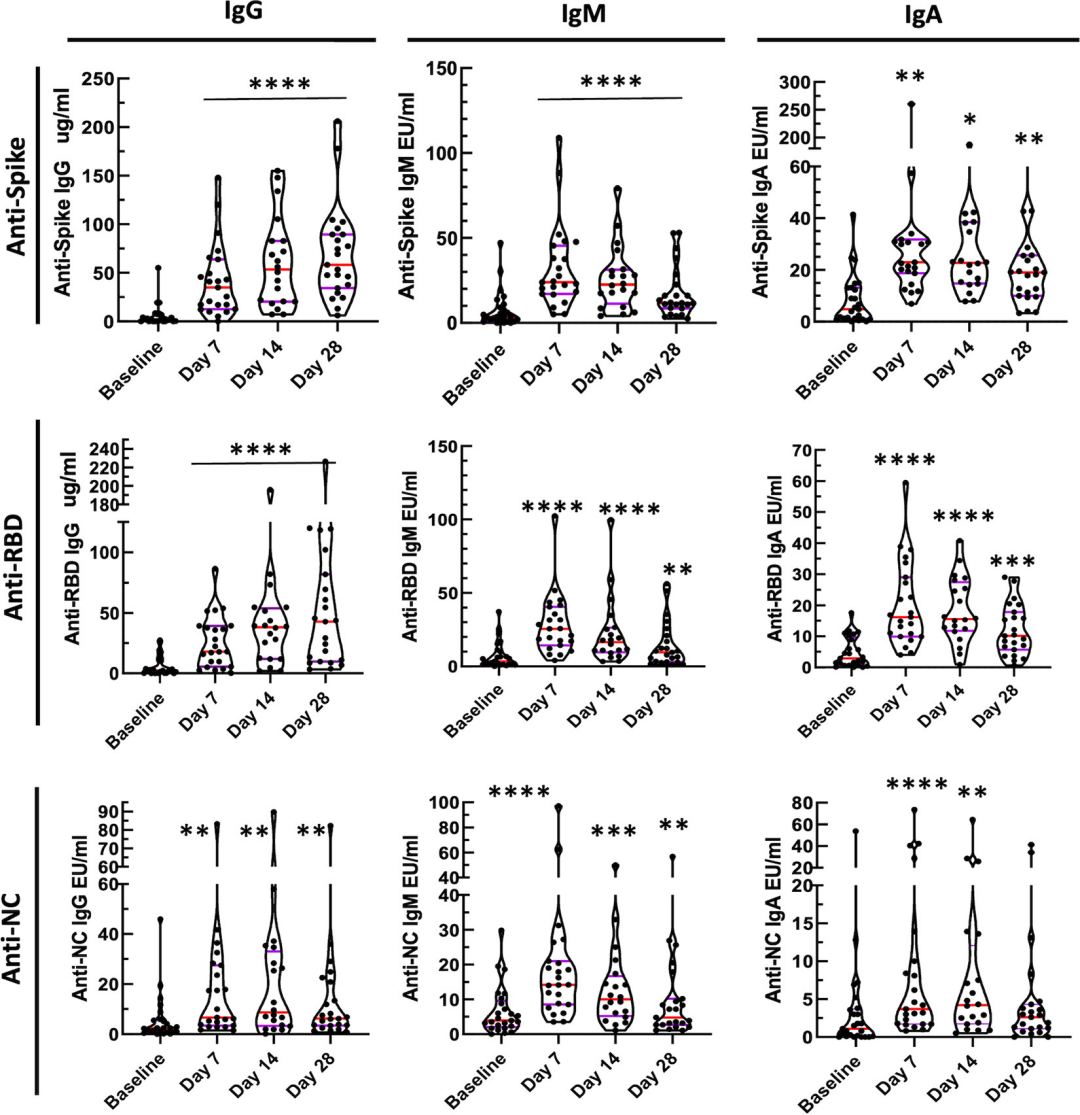
Specific IgG, IgM, and IgA antibodies binding to spike (S), receptor binding domain (RBD), nucleocapsid (NC). Blood was collected on CIP treated participants (*n* = 25) immediately prior to infusion (baseline), and then 7, 14, and 28 days post-infusion. Levels of specific anti-SARS-CoV-2 antibodies were measured and compared to baseline. Paired Wilcoxon rank sum *P* values: * < 0.02; ** < 0.01; *** < 0.001; **** < 0.0001. Medians and the 25 and 75 quartiles are indicated on each violin plot.

**(ii) Levels of specific antibodies in participants.** In order to determine the kinetic pattern of specific SARS-CoV-2 IgG, IgM, and IgA responses to S, RBD, and NC after CIP transfusion, specific levels of IgG, IgM, and IgA were measured at baseline, and on days 7, 14, and 28 after CIP transfusion. All classes of antibodies to the three SARS-CoV-2 target proteins were significantly increased at days 7 and 14 post-transfusion compared to baseline (Fig. 3, *P* < 0.01). Anti-NC IgA levels were reduced at day 28. Table S2 summarizes the distribution for all specific antibodies measured over time.

The median anti-spike IgG, IgM, and IgA levels were significantly increased over baseline at days 7, 14, and 28 (*P* < 0.0001). The median IgM anti-S response was markedly elevated at days 7 and 14 but decreased by day 28. The median anti-RBD IgG, IgM, and IgA levels rose significantly (*P* < 0.0001) with IgG anti-RBD levels that persisted at day 28 while IgM levels began to decrease by day 14 with significant reduction but clearly detectable levels by day 28. Correlation analyses were performed testing the relationship between CIP and post-transfusion circulating specific antibody levels at day 7, but no significant associations were found. Analyses to test the association of CIP specific antibody levels with time to ICU transfer and time to viral PCR negativity were also performed. Only one significant association was found (Table S3 in the supplemental material): a positive one between CIP anti-RBD IgM and time to ICU transfer. This result is interpreted with caution since it was a single ICU case for which the CIP anti-RBD IgM level was markedly elevated. Finally, while it is hypothesized that patients with low circulating antibody titers at baseline may be more likely to benefit from CIP, we are unable to test this in this single-arm study. We did however perform univariate regression analyses for baseline circulating antibody titers and time to ICU and no significant association was found (Table S4).

### Respiratory tract viral clearance.

**FIG 4:**
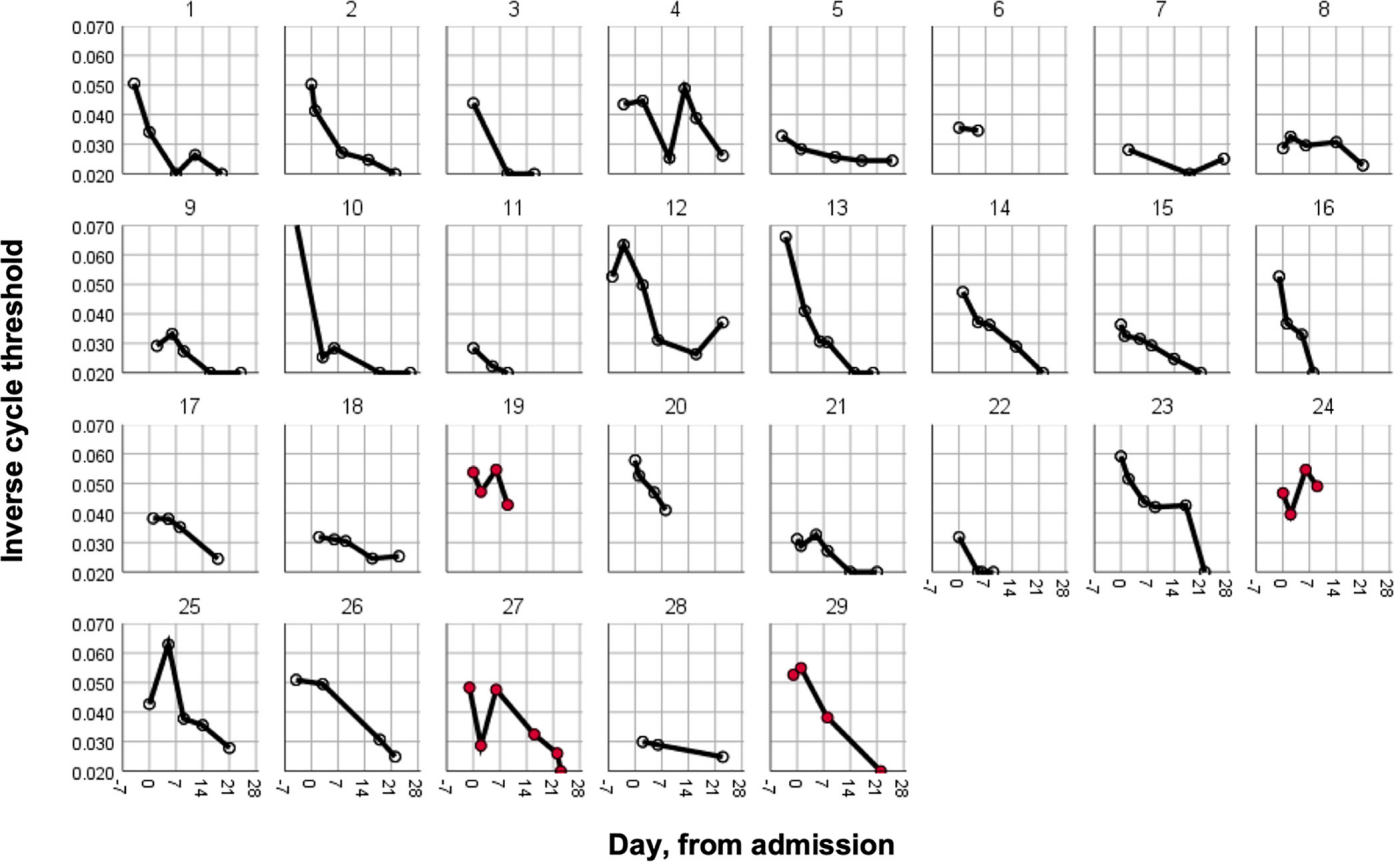
Respiratory tract viral clearance. Serial respiratory tract swabs were collected at baseline and then 4, 7, 14, and 21 days post CIP transfusion. The inverse cycle thresholds for SARS-CoV-2 RNA are graphed. Participants who were ultimately transferred to the ICU are depicted in red.

In order to better understand the kinetics of respiratory tract viral clearance, nasopharyngeal swabs were collected at baseline and days 4, 7, 14, and 21 post-transfusion. SARS-CoV-2 PCR cycle thresholds over time for transfused patients are represented in Fig. 4, with those patients transferring to the ICU denoted in red. Kaplan-Meier survival curves showed a statistically non-significant reduction in time to first negative PCR with CIP compared with 19 controls with >1 PCR test following admission (mean 20.4 versus 24.8 days, log-rank test *P* = 0.22, Fig. S3 in the supplemental material). There was no association between CIP or baseline circulating anti-SARS-CoV-2 specific antibody levels and time-to-PCR negativity (Tables S5 and 6).

Understanding the clinical factors which influence viral clearance may also provide insight into disease pathogenesis and mechanisms of recovery, regardless of the effect of CIP. Therefore, we used these data to test for associations between clinical variables and time-to-PCR negativity (Table S7 in the supplemental material). Of note, hypertension and Angiotensin Converting Enzyme Inhibitor (ACEI) use were the only variables significantly associated with a longer time to negativity.

## DISCUSSION

In this study we report the effect of CIP on the progression to critical illness in people with severe COVID-19. To our knowledge this is the first formal phase 2 study of its kind to target progression to critical illness in hospitalized, non-critically ill patients, and to provide a detailed analysis of the kinetics of anti-SARS-CoV-2 specific antibody levels and viral shedding following CIP infusion. Similar to prior reports, CIP transfusion was well-tolerated(14), with no adverse events attributable to transfusion. This study was implemented at the beginning of the pandemic when estimates for effect size were limited by early available epidemiologic data suggesting that roughly 50% of admitted patients necessitated ICU transfer. Ultimately, this phase 2 single-arm study was underpowered for the primary endpoint of ICU transfer, as local baseline rates of ICU transfer proved to be lower than initially predicted for the population admitted. Kaplan-Meier curves trend in the direction of CIP infusions benefiting the participants. Based on our observed control ICU transfer rate of 27%, a subsequent study in a similar population would require approximately 166 patients per group (N = 332 total) to have 80% power to detect a 50% reduction in ICU transfer.

Anti-SARS-CoV-2 specific IgG, IgM, and IgA antibodies rose to S, RBD, and NC from baseline to post-transfusion, with highly significant differences between baseline and days 7 and 14 post-transfusion. There was little change in IgG anti-S and anti-RBD between day 7 and day 28 post-infusion in most cases, suggesting that transfused antibodies persisted while endogenous antibodies were being made. In contrast, the reductions in specific IgM levels to the various proteins suggests that IgM to IgG switching was occurring leading to rapid decreases in IgM anti-S and anti-RBD antibody levels by day 28. These kinetics differ significantly from that reported elsewhere for non-transfused patients in whom peak IgG levels without CIP occur at 3–4 weeks postinfection (15) and suggest that CIP infusions increased early post-transfusion circulating anti-SARS-CoV-2 specific antibody levels.

In support of our findings that CIP primarily improved SARS-CoV-2 specific antibodies profiles early after infusion, in a small case series of 5 critically ill patients, Shen et al. (16) showed that RBD specific IgG and IgM and neutralizing antibody titers rose between baseline and day 3 after CIP infusions and remained at the same levels through day 7. Similarly, in a study of CIP in older adults with severe COVID-19, Libster et al. reported a significant difference in circulating anti-SARS-CoV-2 spike IgG levels between placebo and CIP infused patients at 24 h post-infusion (17). The quantitation of specific levels of monoclonal antibodies in patients who receive monoclonal antibodies that could be tracked with an anti-idiotypic antibody would provide further insight into persistence of passive antibody infusions and the amounts of endogenously produced specific antibodies. Additionally, future studies examining antibody secretion *in vitro* from B cells isolated from CIP transfused participants could also provide insights into the relative contributions of exogenous and endogenous antibodies.

We did not find a relationship between CIP and post-transfusion circulating specific antibody levels at day 7, likely reflecting variability in CIP antibody levels and function, variable decay of the infused antibodies, and variable rates of endogenous production. We also did not observe a statistically significant effect of CIP on viral clearance, which again may be reflective of variable contributions of host antiviral immunity (be that cellular or humoral) and CIP. Unrelated to CIP transfusion, patients with hypertension or those on an ACE inhibitor had significantly longer time to PCR negativity, which is in keeping with other reports in the literature (18). One proposed explanation for this could be upregulation of ACE2 receptors in these patients, the cellular target of SARS-CoV-2. In addition, dexamethasone use was found to be significantly associated with risk of ICU transfer likely reflective of the more severely ill patients receiving dexamethasone, consistent with local standard practice following the results of the RECOVERY trial (19).

Two years into the pandemic, the evidence for the use of CIP in COVID-19 remains mixed. In favor of CIP, retrospective early studies and analyses of large data sets support its use. For example, the retrospective study by Liu et al. found a benefit of CIP in inpatients who were early in their disease course (symptom onset less than or equal to 7 days prior to admission), and those who were not intubated (20). In the largest data set available from the EAP, earlier administration was associated with improved outcomes compared to late, and transfusion of high titer plasma in non-intubated patients was associated with improved mortality when compared with low titer (21). Additional retrospective evidence suggests that patients with hematologic malignancies specifically may benefit, likely reflective of their relatively immunosuppressed state (22). In a randomized trial focusing on inpatient adults with mild disease who were greater than 75 years of age or 65 with comorbid conditions, Libster et al. found that CIP was protective of progression to severe disease (17). Finally, an analysis of CIP usage and mortality across the pandemic showed an inverse correlation, suggesting that CIP saves lives (23). In contrast, several randomized controlled trials have either terminated early due to low enrollment or found no benefit of CIP (13, 24–26). Many of these trials showed similar signals for benefit but fell short in demonstrating significant clinical benefit as additional standards of care evolved along with pandemic epidemiology.

A summary of the evidence for CIP including the results from the current study support three key factors in its efficacy: timing, product, and patient. That is, early administration of high titer CIP in specific patient populations is likely beneficial in limiting progression to severe disease and/or death. Our study included a significant portion of patients earlier in their disease course, with an average time from symptom onset to admission of 5 days. A larger fraction of patients also did not require oxygen, possibly reflective of a lower severity of illness compared to many other inpatient studies (27), and this may provide further guidance on the target population for treatment or future studies. We also transfused two units rather than one, thereby providing a higher dose and perhaps allowing for a greater clinical effect than in other trials. Of note, an important socioeconomic feature of our study is that it included a majority of participants that identified as Black race or Hispanic ethnicity, groups which are historically underrepresented in clinical trials and have been disproportionately affected by the COVID-19 pandemic. Similar inclusion of representative groups in future studies will be critical to understanding the generalizability of the effects of CIP on COVID-19 disease progression (28, 29).

As this pandemic continues to evolve through subsequent waves, so too must the treatments. The emergence of new more virulent strains of SARS-CoV-2 such as the delta and omicron variants raises the specter of potential resistance to developing therapies, including vaccines and specific monoclonal antibodies (30). In this setting, evidence suggests that polyclonal CIP with its array of antibodies targeting different viral proteins remains an available and importantly adapting therapeutic alternative, particularly early in the disease course.

### Limitations.

This study has several important limitations. Notably, this is a single-arm, single site study with a modest sample size which limits the ability to perform multivariable analysis and statistical modeling. The single-arm prospective nature with retrospective controls also introduces the possibility of selection bias. Additionally, while we present comprehensive antibody titer data for transfused patients, we do not currently have comparable samples from non-transfused control patients for direct comparison to assess the effect of convalescent plasma on circulating antibody levels.

## MATERIALS AND METHODS

The Convalescent Immune Plasma-Treatment (CIP-T) trial is a single-arm prospective, phase II study of CIP for the treatment of severe COVID-19 (hospitalized mild/moderately-ill, non-ICU, meeting World Health Organization (WHO) Ordinal Scale of 3 [hospitalized, no oxygen therapy] or 4 [hospitalized, oxygen by mask or nasal prongs] on the 8-point scale) (31) patients compared to contemporaneous controls which were identified retrospectively, with primary endpoints of 28 day mortality and ICU admission. This study was performed at the University of Virginia Hospital, an academic medical center with a geographically large referral base. Prescreened, high-titer convalescent plasma was donated by the New York Blood Center. Randomized placebo controls were deferred due to the power limitations of a single site study and considering early limited evidence suggesting potential benefit in COVID-19 infection as well as the FDA EAP, which was active at the time of study initiation. An enrollment target of 29 patients was calculated to provide 80% power to detect a 50% reduction in the primary outcome using early historical targets of ICU admission from reports in China and elsewhere (32, 33). This study received approval from the University of Virginia Institutional Review Board (#200114), was conducted under the FDA IND BB-20867, and registered at clinicaltrials.gov, NCT04374565.

### Convalescent plasma.

CIP was provided by the New York Blood Center. Units were prescreened by semi-quantitative IgG to the nucleocapsid protein using the Abbott SARS-CoV-2 chemiluminescence enzyme immunoassay (Architect i2000 chemiluminescent microparticle assay; Abbott; Abbott Park, IL). Per manufacturer, a signal to cutoff (S/CO) value of 1.4 or greater was used to define presence of antibody; the range of S/CO values among CIP units transfused was 2.3 to 9.3. Notably, the use of the Abbott assay, and its S/CO cutoff, precedes the FDA’s recommendation within the EUA that CIP be screened for “high titer” anti-spike levels using the Ortho VITROS SARS-CoV-2 IgG assay (Ortho S/CO ≥ 12), but titers to these targets have been shown to correlate (34, 35). CIP was administered as two paired units from the same donor, when available, (∼220 mL each) at institutional standard transfusion rates over 1–2 days, with the first unit started within 72 h of admission. Prior to subject transfusion, aliquots of CIP units were taken and stored at 4°C for testing ELISA specific IgG, IgM, and IgA to the spike (S), receptor binding domain (RBD) nucleocapsid (NC) proteins.

### ELISA assays for specific IgG, IgM, and IgA for S, RBD, and NC.

Human anti-SARS-CoV2 (anti-spike, anti-RBD, and anti-NC) IgA, IgG, and IgM antibodies were detected by ELISA. Briefly, the plates were coated with 2 μg/mL of recombinant SARS-CoV-2 Protein, S1 Subunit (RayBiotech, # 97-087 Lot: 25056-2004), recombinant NC protein (ProSci, #97-085 Lot: 25067-2004) and recombinant RBD protein (ProSci, #230-30162 Lot: 05U22020TWB) in PBS. Serum antibody levels of IgA, IgG and IgM were detected by HRP conjugated-anti-human IgA antibody, -anti-human IgG antibody, and -anti-human IgM antibody, respectively, at 1:40,000 dilution. Antibody levels were reported as μg/mL of anti-spike and anti-RBD for IgG and ELISA Units of anti-NC IgG, and anti-spike, anti-RBD and anti-NC IgA and IgM antibodies. CIP and patient samples from different time points were processed, stored, and tested in the same manner with reference controls used to normalize the values between runs and samples.

### Study population.

Inclusion criteria were patients >18 years with respiratory symptoms attributable to COVID-19 (confirmed by positive oropharyngeal or nasopharyngeal SARS-CoV-2 PCR testing) within 72 h of admission to an acute care (non-ICU) bed. People who had received chloroquine derivatives were eligible but taken off the drug prior to enrollment. Participants could receive remdesivir and corticosteroids if not part of another anti-COVID-19 trial. Permissive inclusion criteria were set to achieve representative accrual of those with kidney or liver injury, or those with other comorbidities that may have excluded them from other COVID-19 therapeutic trials.

Exclusion criteria were mechanical ventilation or >6 liters per minute nasal cannula oxygen requirement, enrollment into other anti-COVID-19 trials available at our institution (which included the Adaptive COVID-19 Treatment Trials 1 and 2 at that time), prior administration of tocilizumab (anti-interleukin-6 receptor) or siltuximab (anti- interleukin-6), or presence of a preexisting condition, which, in the opinion of the site investigator, could place the individual at substantially increased risk of transfusion-related complications, such as the risk of volume overload with decompensated heart failure, or a history of prior transfusion reaction.

### Controls.

To select contemporaneous controls that could have been feasibly enrolled and treated with CIP in this study, we retrospectively screened adult patients with confirmed COVID-19 who were admitted for >48h during and preceding the study period (April 4–August 16, 2020), and outside of an ICU setting for the first 12 h but who were not enrolled in the treatment arm of this trial. This included patients who were approached for enrollment but declined, and those patients who were not approached at the time of admission for logistical reasons including timing of admission (overnight or on the weekend), and availability of study product. Controls were then reviewed by clinicians with expertise in Pulmonary & Critical Care and/or Infectious Diseases (JMS, SKH, TAT) who were provided History and Physical documentation, but were blinded to patient outcomes. Eligible controls were included in the final analysis if two clinician reviewers agreed the patient met study enrollment criteria including respiratory symptoms attributable to COVID-19 infection. Discordant assessments were adjudicated by a third reviewer. No control patients received convalescent plasma outside of the trial, specifically there was no use of plasma through the EAP.

### Laboratory measurements and clinical follow-up.

At enrollment, demographics, comorbidities and symptoms were collected along with the date of symptom onset. Vital signs were recorded along with oxygen saturation, oxygen requirement, and laboratory values including neutrophil count, lymphocyte count, C-reactive protein, fibrinogen, and d-dimer. Results of chest imaging were recorded but not specifically performed for the trial. For contemporaneous controls, similar data were extracted from the medical record.

Safety assessments were performed on the day of transfusion (baseline), and on days 1, 2, 3, 4, 7, 14, 21, and 28 and 60 post-transfusion. Adverse events were labeled and scored using the Common Terminology Criteria for Adverse Events version 5.0 using the severity scale: 1, Mild: transient or mild discomfort (<48 h); (no medical intervention/therapy required); 2, Moderate: some worsening of symptoms but no or minimal medical intervention/therapy required; 3, Severe: escalation of medical intervention/therapy required; 4, Life-threatening: marked escalation of medical intervention/therapy required; 5, Death (36). The primary endpoint of ICU transfer was considered to have been met for any participant that was physically moved to an ICU-level monitored bed and received critical care as defined by consistent supplemental oxygen of >6 liters per minute at rest, high-flow nasal cannula, noninvasive positive pressure, mechanical ventilation, or vasopressors. In some instances, the participant qualified for ICU transfer based on the above criteria but based on his/her goals level of care was not escalated. These cases were adjudicated as having progressed to critical illness. Serum antibody titers to SARS-CoV-2 total IgG, IgM, and IgA to the spike (S), nucleocapsid (NC) and receptor binding domain (RBD) proteins were completed at baseline and days 7, 14, and 28 post-transfusion. Samples were collected regardless of hospitalization status, and discharged patients were brought back for study visits for data and sample collection. SARS-CoV-2 PCR from nasopharyngeal swabs were performed at baseline and days 4, 7, 14 and 21 for CIP recipients; for contemporaneous controls, all available SARS-CoV-2 PCR results collected for clinical purposes were recorded. PCR assays were performed with either the Xpert Xpress SARS-CoV-2 (Cepheid, Sunnyvale CA, USA), Alinity mSARS-CoV-2 (Abbott Molecular, Des Plaines, IL), or Abbott M2000 RealTime SARS-CoV-2 assay (Abbott, Chicago, IL) using reagents and protocols according to the Emergency Use Authorization approved assays for SARS CoV-2 detection. When assessing cycle thresholds (CT), results performed on the M2000 platform were adjusted by 10 cycles to ensure comparability between platforms. When needed, the lower of the two measurements (i.e., CT to detect the N2 gene targets or CT to detect the E gene targets) was used.

### Statistical analyses.

Descriptive statistics for cohort demographic variables were calculated as reported in the results. To compare patients who received CIP versus controls, Wilcoxon rank-sum test was used for continuous variables and Fisher’s exact test was used for categorical variables. For time-to-event data, Kaplan-Meier curves were generated and compared with log-rank test. For the primary endpoint of mortality, due to the low number of events a univariate Cox regression was performed for CIP as well as other relevant variables of interest which included age, sex, hypertension, diabetes, remdesivir, dexamethasone, BMI, and obesity (defined as BMI ≥30). Similarly, univariate Cox regression analyses were performed for time-to-ICU data. Multivariable Cox regression analysis was also performed to explore the association between CIP and time-to-ICU adjusting for above-mentioned relevant variables. Similar analyses were carried out for time-to-negative PCR. To compare the change of specific antibody measurements following CIP transfusion from baseline, paired non-parametric method Wilcoxon signed rank test was applied for each follow-up date, respectively. Pearson correlation coefficients were calculated and tested for the correlation between CIP and post-transfusion circulating antibody levels.
