# Supplementary material for: Convalescent Plasma for Preventing Critical Illness in COVID-19: a Phase 2 Trial and Immune Profile
Source: Microbiol Spectr. 2022 Feb 23;10(1):e02560-21. doi: 10.1128/spectrum.02560-21 (PMC8865433; doi:10.1128/spectrum.02560-21)
Supplement: SUPPLEMENTAL FILE 1 — Supplemental material. Download SPECTRUM02560-21_Supp_1_seq4.pdf, PDF file, 0.9 MB [file spectrum02560-21_supp_1_seq4.pdf]

SUPPLEMENTAL MATERIAL

SUPPLEMENTAL TABLES

Supplemental Table 1. Participants reporting at least 1 adverse event.

Adverse Event	Convalescent Plasma Recipients (N=29)
Any event, n (%)*	11 (38)
Related adverse event	4 (14)
Severe (≥ grade 3)	8 (28)
Life-threatening (grade 4)	3 (10)
Any serious adverse event	4 (14)
Related serious adverse event	0
Severe (≥ grade 3)	4 (14)
Life-threatening (grade 4)	3 (10)
Death	2 (7)

* Unique adverse events included pulmonary edema (n=6 occurrences in 5 participants), acute respiratory failure (n=4), fever (n=3), hypoxia (n=1) elevated aspartate aminotransferase (n=1), elevated alanine aminotransferase (n=1) atrial fibrillation with rapid ventricular response (n=1), elevated creatinine (n=1), epistaxis (n=1), hypertensive urgency (n=1), hypotension (n=1), sepsis (n=1), worsening sepsis (n=1), syncope (n=1).

Supplemental Table 2. Comparison of specific antibodies in CIP recipients over time.

		Day 0	Day 7		Day 14		Day 28	
				p value		p value		p value
**Spike**	**IgG (µg/ml)**	**1.9** (0.065-6.6)	**35.0** (13.0-64.0)	<0.0001	**53.0** (20.0-83.0)	<0.0001	**58.0** (34.0-90.0)	<0.0001
	**IgM (EU/ml)**	**3.4** (1.45-10.0)	**23.9** (17.10-45.4)	<0.0001	**22.55** (11.33-31.33)	<0.0001	**11.70** (8.5-22.50)	0.0002
	**IgA (EU/ml)**	**4.8** (1.15-13.15)	**23.0** (18.7-31.8)	<0.0001	**22.75** (14.78-38.5)	<0.0001	**19.0** (10.0-25.7)	<0.0001
**RBD**	**IgG (µg/ml)**	**2.26** (0.28-10.8)	**17.9** (5.54-39.13)	<0.0001	**38.1** (12.02-53.87)	<0.0001	**42.8** (9.68-81.82)	<0.0001
	**IgM (EU/ml)**	**3.4** (1.4-10.6)	**25.6** (14.3-40.6)	<0.0001	**16.4** (9.6-26.25)	<0.0001	**9.6** (3.1-20.9)	0.0037
	**IgA (EU/ml)**	**2.9** (0.7-9.6)	**16.2** (9.9-29.0)	<0.0001	**15.5** (11.73-27.43)	<0.0001	**10.1** (5.7-17.8)	0.0002
**NC**	**IgG (EU/ml)**	**2.4** (0.9-5.7)	**6.7** (3.4-27.50)	0.0022	**8.7** (3.3-33.13)	0.0067	**6.2** (3.4-20.90)	0.0033
	**IgM (EU/ml)**	**3.9** (2.0-9.55)	**14.2** (8.5-21.0)	<0.0001	**10.0** (5.2-16.68)	0.0005	**4.8** (2.7-10.2)	0.0079
	**IgA (EU/ml)**	**1.1** (0.1-3.7)	**3.7** (1.7-10.0)	<0.0001	**4.2** (1.75-12.08)	0.0024	**2.6** (1.1-4.3)	0.6320

Wilcoxon rank sum was used to compare baseline levels with day 7, 14, and 28 levels of each specific antibody. Mean and range are listed. n = 25.

Supplemental Table 3. Univariate Cox regression analysis examining the correlation between CIP antibody levels and time to ICU transfer.

Variable	Hazard Ratio (HR)	Std.Err.	z	P value	Lower 95% CI of HR	Upper 95% CI of HR
IgG Spike CIP	1.014	0.01521	0.911	0.362	0.984	1.044
IgG RBD CIP	1.008	0.02256	0.371	0.711	0.965	1.054
IgG NC CIP	0.949	0.261	-0.192	0.848	0.553	1.626
IgM Spike CIP	1.017	0.01451	1.206	0.228	0.989	1.046
IgM RBD CIP	1.144	0.06807	2.254	0.024*	1.018	1.285
IgM NC CIP	1.190	0.1885	1.098	0.272	0.872	1.623
IgA Spike CIP	1.042	0.04789	0.901	0.367	0.953	1.140
IgA RBD CIP	1.058	0.08371	0.711	0.477	0.906	1.235
IgA NC CIP	1.920	1.016	1.234	0.217	0.681	5.415

Levels of all SARS-CoV-2-specific antibodies measured in CIP were tested for association with time to ICU transfer. N=25. Std. Err = standard error.

Supplemental Table 4. Univariate Cox regression analysis for baseline antibody titers and time to ICU.

Variables	Hazard Ratio (HR)	Std. Err.	z	P-value	Lower 95% CI of HR	Upper 95% CI of HR
IgG Spike	0.922	0.142	-0.530	0.599	0.682	1.248
IgM Spike	0.945	0.096	-0.560	0.578	0.775	1.153
IgA Spike	0.910	0.106	-0.810	0.417	0.725	1.143
IgG RBD	1.041	0.060	0.690	0.488	0.929	1.167
IgM RBD	0.895	0.129	-0.770	0.439	0.675	1.186
IgA RBD	0.747	0.209	-1.040	0.296	0.431	1.292
IgG NC	0.918	0.148	-0.530	0.597	0.670	1.258
IgM NC	0.850	0.159	-0.870	0.385	0.589	1.226
IgA NC	0.447	0.364	-0.990	0.323	0.090	2.209

Levels of all SARS-CoV-2-specific antibodies measured from patient plasma at baseline, prior to CIP infusion, were tested for association with time to ICU transfer. N=25. Std. Err = standard error.

Supplemental Table 5: Univariate Cox regression analysis examining the correlation between CIP antibody levels and time to PCR negativity.

Variable	Hazard Ratio (HR)	Std.Err.	z	P value	Lower 95% CI of HR	Upper 95% CI of HR
IgG Spike CIP	1.014	0.009	1.554	0.120	0.996	1.033
IgG RBD CIP	1.006	0.015	0.423	0.672	0.978	1.036
IgG NC CIP	0.924	0.116	-0.635	0.526	0.723	1.181
IgM Spike CIP	0.970	0.038	-0.785	0.432	0.899	1.047
IgM RBD CIP	0.993	0.044	-0.148	0.882	0.910	1.084
IgM NC CIP	0.950	0.091	-0.533	0.594	0.788	1.146
IgA Spike CIP	0.964	0.033	-1.075	0.283	0.901	1.031
IgA RBD CIP	1.056	0.043	1.343	0.179	0.975	1.144
IgA NC CIP	0.701	0.244	-1.022	0.307	0.355	1.385

Levels of all SARS-CoV-2-specific antibodies measured in CIP were tested for association with time to ICU transfer. N=25. Std. Err = standard error.

Supplemental Table 6. Univariate Cox regression analysis for baseline antibody titers and time to PCR negativity.

Variables	Hazard Ratio (HR)	Std. Err.	z	P-value	Lower 95% CI of HR	Upper 95% CI of HR
IgG Spike	1.016	0.035	0.460	0.646	0.950	1.086
IgM Spike	1.005	0.031	0.160	0.872	0.947	1.067
IgA Spike	1.003	0.032	0.090	0.931	0.941	1.069
IgG RBD	0.957	0.044	-0.960	0.338	0.875	1.047
IgM RBD	1.013	0.034	0.390	0.698	0.949	1.081
IgA RBD	0.918	0.064	-1.230	0.218	0.801	1.052
IgG NC	0.920	0.063	-1.220	0.223	0.805	1.052
IgM NC	0.949	0.049	-1.010	0.312	0.857	1.051
IgA NC	0.876	0.100	-1.160	0.248	0.700	1.097

Levels of all SARS-CoV-2-specific antibodies measured from patient plasma at baseline, prior to CIP infusion, were tested for association with time to ICU transfer. N=25. Std. Err = standard error.

Supplemental Table 7. Cox regression analyses for time to negative PCR result.

A. Univariate Cox regression analysis for time to negative PCR (N=48)						
Variables	Hazard Ratio	Std. Err.	z	P>z	Lower 95% CI of HR	Upper 95% CI of HR
CIPT	1.672	0.710	1.210	0.226	0.727	3.843
age	0.988	0.014	-0.810	0.419	0.961	1.017
sex (F vs. M)	1.447	0.573	0.930	0.351	0.666	3.143
hypertension	0.274	0.114	-3.120	0.002	0.122	0.619
diabetes	0.944	0.391	-0.140	0.889	0.419	2.126
BMI	0.994	0.018	-0.360	0.718	0.959	1.029
obese	1.925	1.065	1.180	0.237	0.651	5.694
remdesivir	0.650	0.288	-0.970	0.330	0.273	1.548
dexamethasone	0.569	0.222	-1.450	0.148	0.265	1.222
ACE inhibitor use	0.298	0.141	-2.560	0.011	0.118	0.753
B. Multivariate Cox regression analysis for time to negative PCR (N=45)						
Variables	Hazard Ratio	Std. Err.	z	P>z	Lower 95% CI of HR	Upper 95% CI of HR
CIPT	0.597	0.309	-0.990	0.320	0.216	1.649
age	0.991	0.018	-0.480	0.632	0.956	1.028
Sex (F vs. M)	1.247	0.548	0.500	0.616	0.527	2.949
hypertension	0.244	0.139	-2.480	0.013	0.080	0.743
diabetes	2.910	1.844	1.690	0.092	0.841	10.073
ACE inhibitor use	0.295	0.174	-2.070	0.038	0.093	0.936

Univariate (A) and multivariate (B) cox regression analyses were performed to determine the potential association of clinical variables with time to negative PCR result. Std. Err. = standard error. N=48 for univariate analyses, N=45 for multivariate analysis.

SUPPLEMENTAL FIGURES

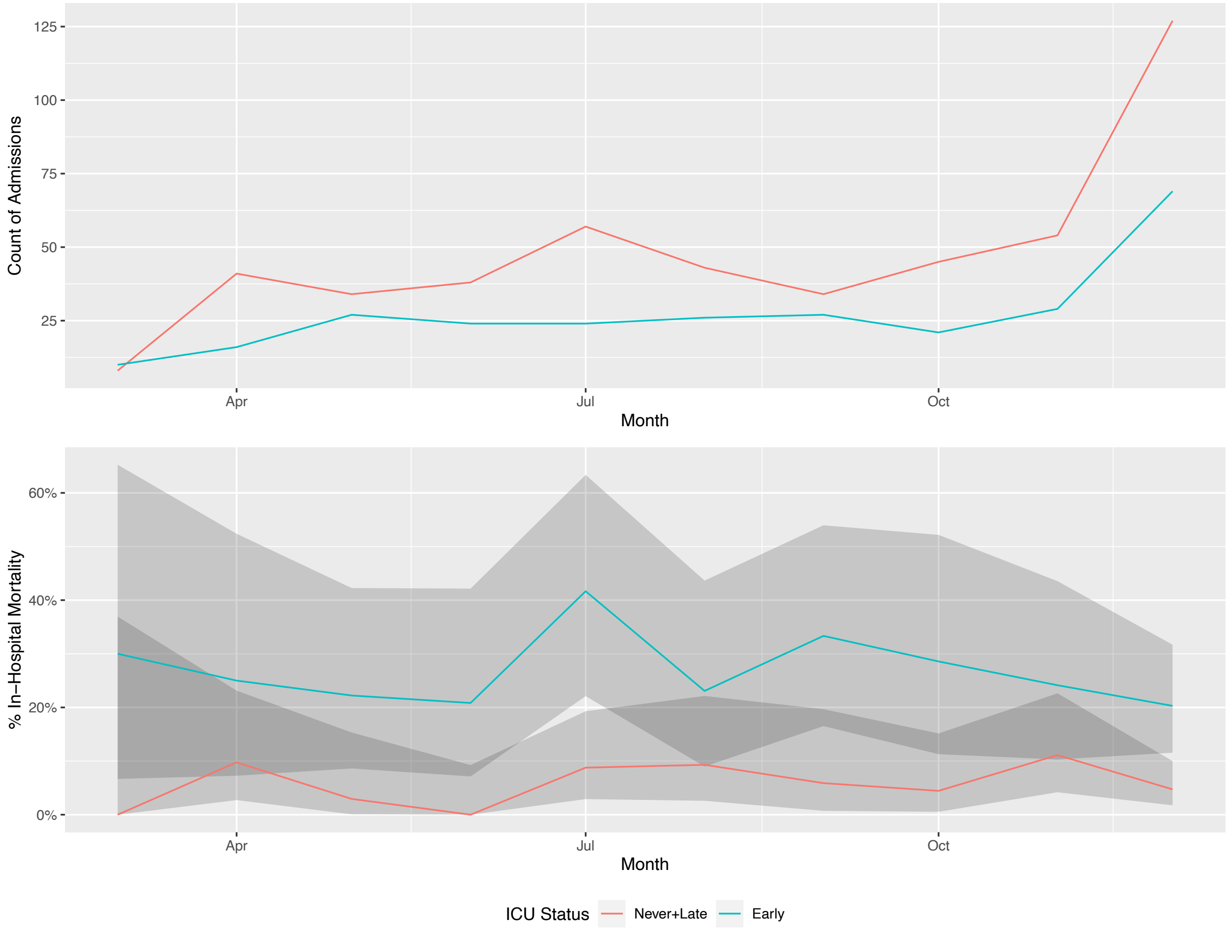

Supplemental Figure 1: COVID-19 case counts and in-hospital mortality throughout the study period in 2020 as determined by extraction from the clinical data warehouse. ICU status never + late refers to cases admitted to a floor bed and either never transferred to the ICU or transferred greater than 24 hours after admission. ICU status early refers to those cases admitted directly to the ICU or transferred to the ICU within 24 hours of admission. Gray bands indicate 95% confidence intervals. Chi-squared test for mortality as determined by month of admission, p=0.4523.

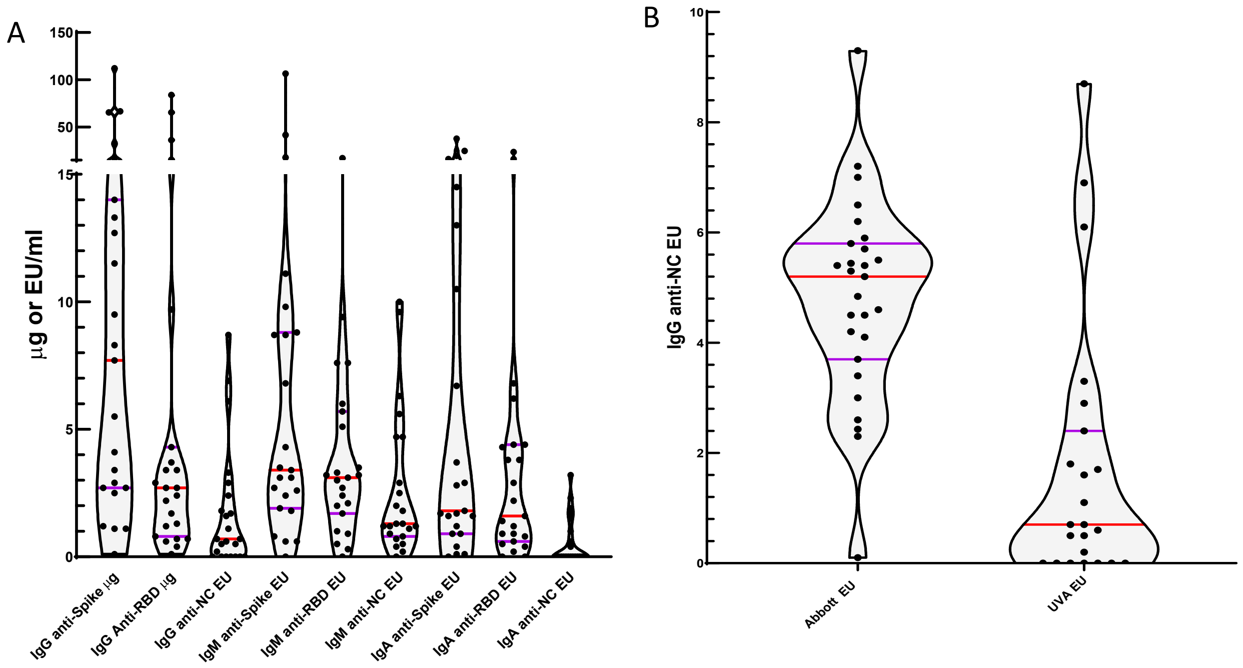

Supplemental Figure 2: Levels of Specific antibodies in CIP (n=28). A) Levels (μg or ELISA Units(EU)/ml) in UVA assays for IgG, IgM, and IgA anti-spike, receptor binding domain (RBD) , and nucleocapsid (NC). B) Levels of IgG anti-NC in Abbott assay with signal/cut-off (S/CO) values and UVA ELISA (EU/ml). Medians and 25th and 75th percentiles are shown.

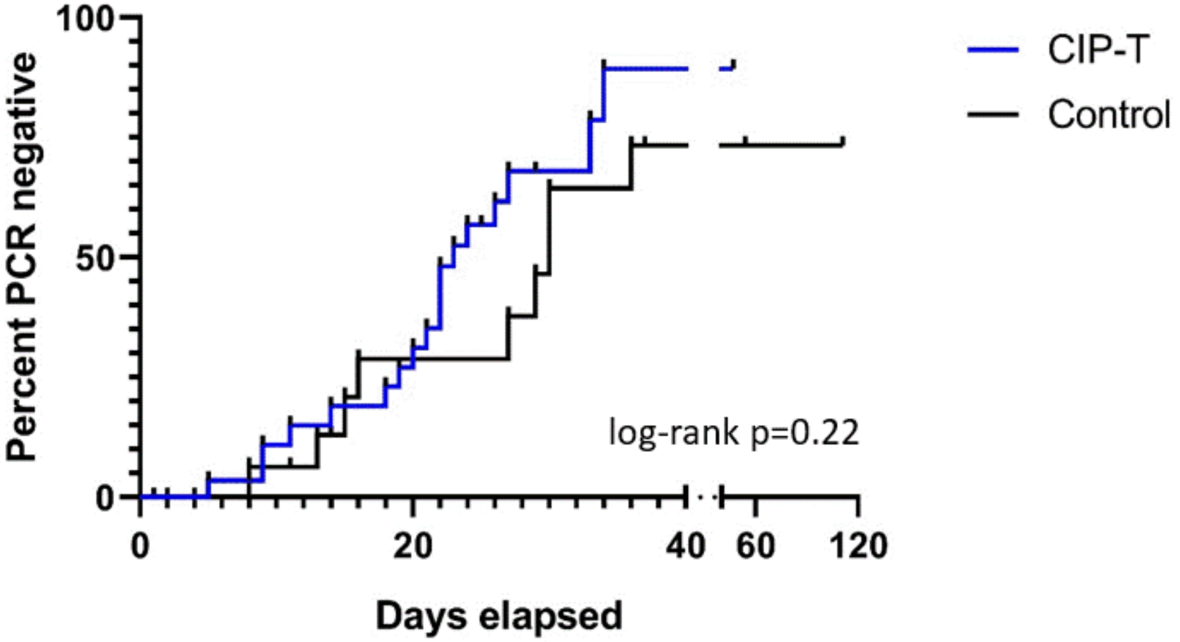

Supplemental Figure 3. Respiratory tract viral clearance. Serial respiratory tract swabs were obtained on CIP participants at baseline, 4, 7, 14, and 21 days post-transfusion. Time to first negative PCR was compared to controls with greater than 1 PCR performed following admission.
